# Cognitive enrichment through art: a randomized controlled trial on the effect of music or visual arts group practice on cognitive and brain development of young children

**DOI:** 10.1186/s12906-024-04433-1

**Published:** 2024-04-04

**Authors:** C. E. James, M. Tingaud, G. Laera, C. Guedj, S. Zuber, R. Diambrini Palazzi, S. Vukovic, J. Richiardi, M. Kliegel, D. Marie

**Affiliations:** 1 University of Applied Sciences and Arts Western Switzerland HES-SO, Geneva School of Health Sciences, Geneva Musical Minds lab (GEMMI lab), Avenue de Champel 47, 1206 Geneva, Switzerland; 2 Faculty of Psychology and Educational Sciences, University of Geneva, Boulevard Carl-Vogt 101, 1205 Geneva, Switzerland; 3 Center for the Interdisciplinary Study of Gerontology and Vulnerability, University of Geneva, Chemin de Pinchat 22, 1227 Carouge (Genève), Switzerland; 4 CIBM Center for Biomedical Imaging, Cognitive and Affective Neuroimaging section, University of Geneva, 1211 Geneva, Switzerland; 5 Accademia d’Archi. Route de Chêne 153, 1224 Chêne-Bougeries, Switzerland; 6 Haute école pédagogique de Vaud (HEP; University of Teacher Education, State of Vaud), Avenue de Cour 33, Lausanne, 1014 Switzerland; 7 Department of Radiology, Lausanne University Hospital and University of Lausanne, Rue du Bugnon 21, Lausanne, 1011 Switzerland; 8 Brain and Behaviour Laboratory, Centre Médical Universitaire, University of Geneva, Rue Michel-Servet 1, Geneva, 1211 Switzerland

**Keywords:** Child development, Executive functions, Randomized controlled trial, Musical instrumental training, Visual arts, Magnetic Resonance Imaging (MRI), Structural connectivity, Task Functional MRI (fMRI), Resting State-fMRI (RS-fMRI), Machine learning

## Abstract

**Background:**

The optimal stimulation for brain development in the early academic years remains unclear. Current research suggests that musical training has a more profound impact on children's executive functions (EF) compared to other art forms. What is crucially lacking is a large-scale, long-term genuine randomized controlled trial (RCT) in cognitive neuroscience, comparing musical instrumental training (MIP) to another art form, and a control group (CG). This study aims to fill this gap by using machine learning to develop a multivariate model that tracks the interconnected brain and EF development during the academic years, with or without music or other art training.

**Methods:**

The study plans to enroll 150 children aged 6-8 years and randomly assign them to three groups: Orchestra in Class (OC), Visual Arts (VA), and a control group (CG). Anticipating a 30% attrition rate, each group aims to retain at least 35 participants. The research consists of three analytical stages: 1) baseline analysis correlating EF, brain data, age, gender, and socioeconomic status, 2) comparison between groups and over time of EF brain and behavioral development and their interactions, including hypothesis testing, and 3) exploratory analysis combining behavioral and brain data. The intervention includes intensive art classes once a week, and incremental home training over two years, with the CG receiving six annual cultural outings.

**Discussion:**

This study examines the potential benefits of intensive group arts education, especially contrasting music with visual arts, on EF development in children. It will investigate how artistic enrichment potentially influences the presumed typical transition from a more unified to a more multifaceted EF structure around age eight, comparing these findings against a minimally enriched active control group. This research could significantly influence the incorporation of intensive art interventions in standard curricula.

**Trial registration:**

The project was accepted after peer-review by the Swiss National Science Foundation (SNSF no. 100014_214977) on March 29, 2023. The study protocol received approval from the Cantonal Commission for Ethics in Human Research of Geneva (CCER, BASEC-ID 2023-01016), which is part of Swiss ethics, on October 25, 2023. The study is registered at clinicaltrials.gov (NCT05912270).

**Supplementary Information:**

The online version contains supplementary material available at 10.1186/s12906-024-04433-1.

## Background

This research proposal is founded on an extensive literature review (see Musical instrumental practice as a developmental driving force) and on a recent behavioral study of our team [1]. This RCT showed broad executive functions’ improvements in the intervention group (Orchestra in Class (OC), practicing string instruments) compared to the active control group (sensitization to music) in initially musically naïve 10-year-old primary school children after two years (see A recent Orchestra in Class (OC) randomized controlled study). Differences were not yet significant after the first year.

To the best of our knowledge, this is the first study that could show such broad benefits of music practice in a class setting compared to an active control group that also learned music. The outcomes suggest that practicing a complex instrument over two years, but also dynamic interactions during ensemble playing, incited these benefits; exact brain correlates of such effects remain unknown.

Concerning Visual Arts (VA), such comparative studies in young children do not exist, apart from the studies comparing music practice to VA as a control group [2–8]. Music practice systematically induced greater cognitive benefits than VA.

The multimodal nature of musical performance may represent one of the explanations of its efficacy as a driving force of learning. Auditory, sensorimotor, proprioceptive, and visual feedback are linked with the expected acoustic musical end product and the music score to correct and refine the performance, interconnected by memory functions, executive functions, and logical thinking/planning.

### Executive Functions (EF)

EF represent an umbrella term for a set of multidimensional abilities that consist in regulating thoughts in the context of goal-directed behavior, implied in both academic and daily activities’ settings, crucial in the context of child cognitive development. EF is multidimensional, but which subfunctions compose EF differs in the literature. Miyake, Snyder et al. [9, 10] divide EF into three core components 1) shifting attention between tasks/cognitive flexibility, 2) working memory (WM) updating and 3) inhibitory control. WM implies storing and manipulating information temporarily, while the information is no longer perceptually present [11]. Some authors include complex higher-order competencies in EF: reasoning, problem-solving and planning, hence abstract thinking [11, 12], supported by strong relationships with the three core EF components.

### Development of EF in childhood

EF components show different developmental trajectories. Although an important maturation occurs between three and eight years-of-age (YOA), corresponding to a significant increase in overall brain size, reaching the adult size at ~eight YOA [13], some components mature into adolescence (e.g. WM). Whereas the early period appears relatively unitary, afterwards individual differences become progressively multidimensional, with WM developing differently after the early years [14]. This observation suggests that further EF development may be influenced by experience. Despite the large literature on EF in children, no truly developmental account of EF and its cerebral substrates across childhood exists, specifically concerning the processes by which young children move from one level to another after the early years [15]. We’ve known for two decades [16] that the prefrontal cortex plays a major role in EF development during childhood. Recent research, however, demonstrated that the role of the prefrontal cortex in EF should be considered within the context of frontoparietal brain network dynamics, also known as the central executive network (CEN), that mediates EF across its different components [17].

Data from structural equation modeling to identify latent factors suggest that executive functions evolve from a more unified model to a more multifaceted framework during the progression from childhood to adulthood [18–20]. Research focusing on children around the age of eight typically reveals a shift from a homogenous system to a dual-factor configuration within this age group [19]. The first dissociation of the singular entity generally shows a separation of the inhibition factor and a WM–flexibility factor [21].

### Musical instrumental practice as a developmental driving force

Musical instrumental practice (MIP) seems more effective in driving cognitive development and associated brain plasticity than other leisure activities [8, 22–25]. This may be explained by 1) the direct involvement of core EF functions in MIP: WM, attention, processing speed, inhibition, and cognitive flexibility may play a role as hubs, supporting higher-order abilities like logical thinking and planning, 2) the observation that making or appreciating music stimulates the dopaminergic system and also hormones from the endocrine system, that influence the reward system [26–28], and may reinforce learning [29], 3) the fact that, of all sensory systems, including vision, the auditory system processes input by far the fastest and therefore is the most computationally intensive neural network [30]. Moreover, multiple musical inputs must be treated simultaneously in a group setting like OC. This ultrafast processing in a dynamic setting may drive the subcomponents of WM updating, i.e. temporal sequencing and monitoring [9].

MIP could provoke better development of cognitive skills and school grades -even when controlling for individual and parental characteristics- compared to sports. Interestingly, these effects were stronger in adolescents from a lower socioeconomic background [31]. When compared to drama courses or no training, MIP and singing incited modest but significant increases in full-scale intelligence quotient (IQ) [22]; moreover, these advantages proved long-lasting [23]. For our view on potential effects of MIP on IQ/general intelligence, see A recent controversy on the impact of musical practice on cognitive child development. Haywood et al. [32] tried to replicate Schellenberg’s results but failed. However, this study [32] severely lacked statistical power and was not peer-reviewed. Compared to sports training or no training, two years of MIP in 6-7-year-old children from low-income families induced an increase in interhemispheric structural connectivity and higher activation of inhibition regions, but no improvement in EF performance [24, 25]. This study of medium scale (n~18 per group) was not an RCT.

#### Comparative impact of musical instrument practice and visual arts training on development

Several studies used visual arts (VA) as an active control condition to compare with music practice training in children. Music training groups systematically showed greater advantages for higher-order cognitive functions [2–7]. A recent longitudinal study compared the influence of extracurricular activities in junior high school students (12-13 YOA) comparing musical to visual arts activities [33]. Both music and visual arts extracurricular activities positively affected academic performance, mediated by improvements in respective art scores. This relationship held even after adjusting for socioeconomic status. The study acknowledged several limitations, including the inability to specify which activities within the arts improve performance and the influence of cultural backgrounds on the impact of arts activities. To the best of our knowledge, no study focused exclusively on VA training and its impact on child development in young primary school children compared to a control group.

Open issues: Perform a* large-scale RCT* in young children with initially no artistic experience, comparing MIP to VA, and to a control group receiving minimal stimulation with respect to the study outcomes.

#### Far transfer effects of musical instrumental training in school-aged children

WM updating [34] plays a crucial role during group music practice, as one has to continuously compare what just sounded with what is coming up. This holds for the sounds produced by the individual player, and the surrounding musical context, but also for visual aspects of the individual’s and fellow-players’ motor behavior [34, 35].

In children, learning to play a complex musical instrument, practicing regularly over extended periods of time, manifests as a driving force for far transfer effects on basic and higher order cognition, sensorimotor skills, and particularly EF [2, 3, 5, 6, 8, 22, 23, 35–48].

Near transfer refers to improved performance directly linked to the trained domain. Far transfer refers to training effects that go beyond the boundaries of the trained domain, i.e. between contexts that seem remote to one another [48].

As summarized in a review by Dumont et al. [49], the literature on music practice in primary school-children is largely inconclusive because of the lack of genuine RCTs comparing music practice to other art forms and a control group. The authors also observed strong heterogeneity across the different studies, concerning group size, intensity, and nature of the music regimens. Of the 46 music intervention studies [49], only two used an RCT design. Neither of these two studies [7, 50] concerned long-term instrumental training on a complex instrument.

An RCT on 6-8-year-old children could show promising effects after eight months of once-weekly musical instrumental training in small groups compared to VA training and a wait-list group [8]. Only the music group progressed for inhibition, visual WM, and selective attention, but comparisons between the two art groups did not reach significance. We consider the training period was too short and not intensive enough to reveal distinct benefits for MIP compared to VA instruction. The study proposed here lasts more than twice as long, the provided lessons are twice as intensive, and finally, extensive neuroimaging is added.

Concerning training in large groups, Rickard et al. [51] investigated school-based instruction in young adolescents over 5-6 months in a pseudo-randomized study and in an RCT. No developmental benefits of music lessons in a class setting manifested, compared to control groups that received drama and art classes. However, the proposed music training [51] did not focus on long-term MIP. Another group setting study -not an RCT- on playing an instrument, compared children (9-11 years), after two years of intensive school music training, with a passive control group [52]. The authors found evidence for enhanced short-term auditory and visual memory in the music groups. Slater et al. [53], in a pseudo-randomized group setting, observed a catch-up in reading performance in low-income children after one year of music training, comprising focused instrumental training. However, they only used a passive control group, and in the music group only children that desired to participate were included, inducing a motivational bias.

Aleman et al. [54] studied “El Sistema” orchestra training in Venezuela in children of 6-14 YOA. Self-control improved, but not cognitive abilities. However, most children trained for less than a year, which is insufficient, two years seem required to show important cognitive changes [1, 55]. In the first year, most children merely received recorder lessons, a rudimentary musical instrument, or none at all [54].

Jaschke et al. [3] performed an RCT contrasting music vs arts programs for two and a half years in young primary school children. Music training consisted of enriched sensitization to music and not focused instrumental training. The results were tainted by adding a second group of children taking private music lessons outside of the randomization procedure. The two music training groups were confounded in most analyses. Inhibition, planning, and verbal IQ improved in the two music groups compared to arts and passive control. The VA group outperformed the music groups in visuospatial memory tests.

Open issues: Given the current state of research in the field, we conclude that long-term learning to play a complex musical instrument is the driving force for far transfer to cognitive processes in childhood, and not just any kind of music instruction. Genuine RCTs are mandatory to exclude motivational bias.

#### Confounding effects when studying transfer effects from musical training to other domains

Few studies investigated children with low or variable socio-economic status [1, 25, 56], although those children may benefit more from musical training [31, 53]. Evidence of beneficial musical practice effects on child development predominantly concerns children of parents with a high socio-economic background [57] and typically results from private lessons. High-functioning children may be more interested in learning to play a musical instrument, inducing a motivational advantage [58]. Personality traits of parents and children, and the child’s drive and instrument preference, may strongly impact learning and subsequent transfer to other cognitive skills [57, 59, 60].

Open issues: Evaluate and contrast the effects of MIP with other forms of artistic training and control groups in young children without prior music experience, with variable socio-economic backgrounds.

#### A recent Orchestra in Class (OC) randomized controlled study

Our team recently performed an RCT that demonstrated for the first time that focused MIP in a group setting, as compared to traditional sensitization to music, can provoke robust cognitive and sensorimotor transfer effects [1]. Over the last two years of primary school (at start ~10 YOA), 69 children received musical instruction twice a week by professional musicians within the regular school curriculum. The intervention group learned to play string instruments through OC; the control group, peers in parallel classes, were sensitized to music via listening, theory, moderate practice on simple instruments, and some chorus singing (traditional mandatory Swiss music courses). Broad benefits manifested in the OC group as compared to the control group in terms of musical abilities, sensorimotor hand function and bimanual coordination (near transfer), but also of WM, attention, processing speed, cognitive flexibility, and matrix reasoning (far transfer). Learning to play a complex instrument in a dynamic group setting impacted development much stronger than classical sensitization to music, with the same duration and frequency.

This study on 10-12 years old children by our team is, to the best of our knowledge, the only RCT comparing OC training to sensitization to music, therefore distilling the added value of MIP as compared to more general music lessons, involving active listening, and some moderate practice. However, our study was a cluster RCT^1^; in the current study we propose a genuine RCT with stratified randomization (see Study design), that ensures a similar baseline in all groups.

Several other limitations manifested in our study [1]: 1) EF function was not extensively investigated (only one test per construct), 2) the children were already in advanced childhood, 3) no brain imaging was involved that may underpin and better explain the observed near and far transfer effects. Finally, using an active control group also learning music may have induced overlapping effects, and a passive control group lacked.

Open issues: Perform a genuine large-scale RCT comparing MIP to another art form and a control group in children at the start of their academic curriculum, with an optimal age for brain plasticity and learning, using a large EF psychometric battery and multimodal neuroimaging.

#### Lack of adequate data-driven models explaining transfer effects of OC or other art instruction on brain and behavior and their interrelationships

Solid validated data-driven models based on large-scale empirical data on the cognitive and cerebral scaffolds of musical instrumental group training or other art training are lacking. We hypothesize that far cognitive transfer effects of music training may be explained by the direct involvement of core EF components that may play a role as hubs [1, 61–63] (see Musical instrumental practice as a developmental driving force). We postulate that the transferred skills are not specific to music making, but generic, yet intensively solicited during OC, hence inducing multiple transfer effects and widely distributed functional and structural plasticity.

To comprehensively test these hypotheses, the EF constructs should be subject to multiple testing (see Study design) and associated with plasticity in known brain networks supporting those functions. Moreover, testing these hypotheses by comparing MIP to VA and to an active low-intensity control condition (CG) would strengthen inferences and allow modeling musical instrumental learning but also visual artistic learning with an optimal fit, considering confounding factors.

Open issues: The foreseen multivariate data-driven analyses may uncover hidden relationships, allowing to build data-driven models of OC and VA learning. If validated, we will design and test models that may contribute to numerous fields of study, especially given the lack of understanding about brain developmental mechanisms enabling far-transfer effects of art interventions in young children. The collected data will allow the creation of a data-driven multivariate model of children's interrelated brain and EF development during the first years of their academic curriculum (initially 6-8 years), with or without musical or visual arts enrichment. To our knowledge, such a model does not exist.

#### A recent controversy on the impact of musical practice on cognitive child development

Recently, a controversy occurred concerning the effectiveness of musical practice to provoke far cognitive transfer in children. A meta-analysis on music practice by Sala and Gobet [64] concluded that music training has no positive effect on children's cognitive development. However, this analysis blended very different music training programs, of which a minority involved MIP. The analysis included only post-test designs. Effect sizes were grouped into four broad categories confounding distinct abilities: verbal and non-verbal ability, memory, and speed. Therefore, the study does not allow drawing conclusions on EF.

A subsequent meta-analysis by Román-Caballero et al. [47], invalidated that of Sala and Gobet [64]. Román-Caballero et al. [47] disclosed that only one-third of the studies analyzed by Sala and Gobet [64] were RCTs focused on musical instrumental learning. They could show, targeting children learning to play a musical instrument, a small but meaningful positive effect size (Hedges’ g = 0.26, corrected for small sample bias) for improvement of cognitive skills and academic achievement in short-term MIP studies. Román-Caballero et al. [47] showed that EF, literacy, short-term memory and visuo-spatial skills improved, but not general intelligence, mathematics, phonological processing, processing speed or long-term memory. Long-term studies were too rare to draw conclusions.

We do not consider it plausible that music training influences general intelligence (IQ). Román-Caballero et al. [47] suggest a link in the other direction, in line with Wan and Schlaug [65]: preexisting cognitive advantages and higher levels of academic achievement, such as those observed at baseline in self-selection studies, would favor learning to play a musical instrument. This underlines the need for genuine RCTs and variable socio-economic backgrounds. However, scores of an IQ test, a “proxy” of the latent variable of general intelligence, may slightly increase with improved EF [66].

Another research group led by Bigand [48] also refuted the results by Sala and Gobet [64]. They reanalyzed the included articles and discovered that the conclusions of Sala and Gobet partially depended on the inability to distinguish between near and far transfer. Near-transfer research shouldn't be included in a far-transfer meta-analysis. Correcting the inclusion and exclusion data files used by Sala and Gobet [64], Bigand’s reanalysis resulted in a meaningful effect size for far cognitive transfer, even if different types of musical training remained included [48].

All three meta-analyses [47, 48, 64] pooled data from school-aged children and adolescents (~4 to ~16 YOA), whereas the most sensitive period for learning and particularly so for brain plasticity, may manifest in the early school years (around 7 YOA) [65, 67]. Yet, a recent analysis has shown differing opinions on the ideal age for effective interventions [68] (refer to section "Rationale and overall goals of the project and its interventions").

Finally, of the studies included in all three meta-analyses [47, 48, 64], a minority involved group practice, which could exert a stronger influence on cognitive development due to dynamic interaction [1].

Open issues: contribute to resolving the controversy on the specific effect of group MIP on EF and its cerebral substrates in young children.

## Rationale and overall goals of the project and its interventions

The key purpose of this study is to examine the potential benefits of long-term intensive group interventions “Orchestra in Class” (OC), compared to visual arts training (VA), as additional educational tools, and to no artistic training (active control group (CG)), on cognitive and cerebral development.

The present study crucially extends the available literature on the benefits of musical practice on child development in several major areas: 1) it focuses on intensive training on complex musical instruments, 2) in a class-size orchestra setting, 3) as part of a genuine large-scale RCT, 4) comparing this training not only to visual arts training of equal intensity and setting but also to an active control group without formal artistic training, 3) spanning two full academic years, 4) targeting young children at the onset of their academic education, when behavioral and brain plasticity are at their peak, a period marked by a transition from more unified to more multifaceted EF, 5) using a large psychometric EF battery, 6) in conjunction with cutting-edge multimodal neuroimaging, including brain network dynamics, 7) allowing unified data-driven analyses, via machine learning, enabling the development of a data-driven multivariate model of initially 6-8-year-olds’ interconnected brain and behavioral EF development over two years with or without musical or visual arts enrichment.

In terms of impact, the overall goal is, in case of positive results, more widespread adoption of government-funded musical practice facilities or programs implementing (neuro)scientific findings into the public education system in the Geneva canton and beyond.

Therefore, intervention groups will aim to be class-sized (according to the Swiss educational system). This study investigates whether OC can boost EF and its cerebral substrates, based on a rich but still inconclusive literature [2, 3, 5, 6, 8, 22–24, 35, 37, 39, 40, 42, 44–48, 65].

The usage of four instruments with varying pitch ranges (violin-viola-cello-double bass) will permit a more intricate musical framework (polyphony). Greater independence within the context of polyphony requires more cognitive effort from each musician than if all perform the same melody.

We will compare two distinct art interventions: one in the auditory and one in the visual domain: OC (experimental group 1) versus VA (experimental group 2). Apart from being a second experimental condition, the VA group also allows controlling for the contextual influences of regular stimulating class interventions, and individual art practice at home. This will help clarify the debate regarding the specific impact of group MIP on EF and their underlying brain mechanisms in young children (see A recent controversy on the impact of musical practice on cognitive child development).

We will also acquire full data on an active control group (CG) to control for natural child development in the context of standard education, contextual influences (family, peers), biological maturation, and test-retest effects. To prevent motivational bias during testing, these children will be offered six cultural outings per year, expected to have a minimal impact on the specific outcomes we aim to measure (minimal learning and transfer effects).

To differentiate the impact of OC, we propose to use VA classes, another group art intervention. Two studies used VA as an active control condition for group musical instrumental practice in children; music-training groups demonstrated far greater benefits for cognitive functions [3, 8]. Nevertheless, VA may provoke specific advantages in visuospatial memory tasks [3]. In a recent study [33], both musical and visual arts extracurricular activities positively influenced academic performance in adolescents (12-13 years old), regardless of socioeconomic status. Comparisons between OC, VA and a CG will allow disentangling specific interventional effects of OC and VA on brain and behavior.

Our recent behavioral RCT [1] on children attending public primary schools in popular neighborhoods in Geneva (age ~10 at the start of musical training), compared OC to sensitization to music (active control group). The study showed salient multiple transfer effects in the cognitive and sensorimotor domain following OC after 2 years of training, particularly on EF. We undertook this study to collect preliminary data, aiming to integrate neuroimaging in a larger scale study in the future. The strong behavioral preliminary data support the relevance of the here described RCT. In the study depicted here, we will not use an active control group following sensitization to music again for three reasons. First, we cannot exclude that confounding effects may occur as the control groups also practice music; second, as the children will be randomized individually, and the OC instruction is extracurricular, all children also receive the Swiss standard music lessons. In a recent study that compared piano lessons to sensitization to music (active control group) in healthy older adults, the active control group also showed some learning and brain plasticity effects [69–71].

Executive functions (EF) demonstrated increased malleability during the early school years [72, 73]. Consequently, it is crucial to undertake more detailed research within this age group. Notably, in our prior study [1], the children were approximately 10 years old at the beginning of the intervention. The most sensitive period for learning and brain plasticity, may more strongly show in the early school years (around 7 YOA) [65, 67]. However, a recent analysis highlighted the lack of consensus on the optimal age for interventions to be effective [68]. Our study, with an age range of up to maximum three years at the start within the groups, then following the children over a period of two years during two distinct intensive arts interventions, will allow us to examine the age factor relative to both the timing and content of the interventions’ influence on cognitive and brain plasticity.

To transcend classical top-down approaches, based on a priori hypotheses that we will perform at the start, the ultimate analysis goal is data-driven multivariate analyses. We will use machine learning to evaluate multivariate associations between EF behavior and brain substrates, comparing OC's impact over time to VA interventions and to the CG. Ultimately, modeling will include measures of executive function in daily life settings and academic achievement. These analyses may uncover novel, multifactorial, and subtle aspects of OC's and VA’s impact on child brain and EF development, compared to each other and to the CG [74]. This will allow the development of an integrated model of EF and cerebral development over the first years of the academic curriculum, with and without extracurricular artistic enrichment of two distinct kinds.

## Study objectives

### Hypotheses and outcomes

#### Primary hypothesis and expected outcomes

We anticipate cognitive far transfer effects following OC and, to a lesser degree following VA exhibited by improved EF performance, particularly auditory and visual WM performance during fMRI tasks, as compared to the CG. We expect greater improvement in OC except for visual WM.

#### Secondary hypotheses and expected outcomes

1. We foresee that OC interventions will be associated with increased functional and structural brain plasticity as compared to VA and the CG (OC > VA > CG), notably in a set of temporal (medial and lateral (auditory)), prefrontal, and superior parietal areas, as well as in the basal ganglia (striatum), the cerebellum and the corpus callosum, supporting WM, EF and attention [24, 35, 73, 75].

We also anticipate greater Functional Connectivity (FC) changes following the same gradient (OC > VA > CG) in the Default Mode Network (DMN), Central Executive Network (CEN) and Salience Network (SN) [76, 77], in conjunction with increased or decreased structural connectivity [78]. In addition, plasticity associated with VA might occur specifically in the visual cortex.

2) We hypothesize that we can model child EF development over two years in primary school children (initially 6-8 YOA) using multivariate data-driven analyses (see Statistical analysis plan and sample size calculation Planned statistical analyses, Step 3) that take into account all behavioral and brain data at all three time points, across the three experimental conditions (OC vs. VA vs. CG).

### Primary and secondary endpoints

Primary Endpoint: Improve WM performance through OC and to a lesser extent through VA interventions, as assessed by psychometric testing and fMRI tasks, in primary school children of 6-8 years at the start, following them over two years of intensive OC vs. VA group-interventions, as compared to baseline data (representing progress) and to the CG data (representing natural development). The rationale for the selection of WM was explained in *1.3.2.*

Secondary Endpoints:

1. Identify significant changes in structural and functional brain plasticity, i.e. biomarkers (brain features), comparing 1-year and 2-year values to baseline values (i.e. progress) of both art interventions and to the CG values (natural development).
2. Establish significant correlations between brain plasticity and EF performance change, comprising WM, following the two interventions, particularly MIP (OC), after one and two years of interventions, compared to baseline and to the CG values.
3. Create a comprehensive data-driven model of child interrelated brain and EF development over the first two years of their academic curriculum, with and without additional cognitive enrichment (OC vs. VA vs. CG).
4. Within the context of this comprehensive model of EF, explore how artistic enrichment may influence the typical transition from a more unified to a more multifaceted EF structure around age eight, comparing these findings against a minimally enriched CG [18, 20].

*Baseline factors* that may influence the endpoints are, amongst others, age, gender, socioeconomic background, and WM performance at baseline. These confounding factors will be partially controlled for through the stratified randomization and also in the statistical analyses (by means of covariates).

## Methods and design

### Study design

The proposed study is a 1) randomized (stratified), 2) three-arm, 3) open, 4) monocentric (primary schools in Geneva) 5) national (Switzerland) 6) intervention study.

Stratified randomization will be applied to compose equivalent groups, stratifying for the factors 1) age, 2) gender, and 3) socio-economic background to balance all groups for those factors.

3-arm design: one experimental group (OC), another art group (VA), and an active control group (CG). The study design is open, as the assigned group cannot be blinded. Blinding will only be applied at the randomization level and, when possible, during the statistical analyses.

However, participants in all groups will be ignorant of the more robust hypotheses for brain and behavioral plasticity for the OC and, to a lesser degree for the VA group. We will explain to the parents and their children that we will compare the potential impact on intellectual child development, following two distinct art interventions or cultural outings.

The psychometric battery consists exclusively of validated tests.

A full list of the psychometric battery tests and MRI measurements can be found in Tables 3 and 4 in section "Study procedures with timelines". Importantly, each EF construct is measured by multiple tests.

We will cope with test-retest effects by 1) using different versions, or different items, or different order of items of the tests at T0 (baseline, before the interventions), at T1 (after one year of interventions) and at T2 (after two years of interventions), and 2) by taking into account the test-retest effects in the CG between timepoints.

### Study interventions

#### Intervention procedures

Children receive the interventions in groups or “classes” of ~15-18 children, 1 hour and 30 minutes per week (three OC groups and three VA groups). The interventions will start in March 2024 (wave 1, one group per condition) and in September 2024 (wave 2, two groups per condition). About 50 children will participate in each experimental condition (OC, VA, CG), so 150 children in total. Accounting for an attrition of maximum 30%, final analyses would include a minimum of 35 children per group, ensuring adequate statistical power (see Statistical analysis plan and sample size calculation).

#### Description of interventions

##### Experimental intervention group 1: OC

Diverse musical styles will be applied to offer a varied and attractive program. Two teachers will instruct the class, one for the higher-pitched instruments (violin, viola) and one for the lower-pitched instruments (cello, double bass). First, based on mutual consent, the children choose their instrument (7 violins, 5 violas, 4 cellos, and 2 double basses for 18 children), composing a true string orchestra composed of four instruments with different pitch ranges. At the start, the child will play by bowing open strings smoothly (without using the left-hand fingers), and by plucking the strings with the right hand (pizzicato). Progressively, the fingers of the left hand will be used to stop the strings, first while playing pizzicato, and later in combination with more and more diversely articulated use of the bow (right hand). Meanwhile, the complexity of rhythms will increase gradually. After approximately one month, children take their instruments home and are encouraged to practice 20 minutes per day, five days per week in the 1^st^ year, and 30 minutes in the 2^nd^ year, verified via an online diary. Score reading is introduced in the 1^st^ year but fully applied in the 2^nd^. From the very beginning, the child will be made aware of being part of a polyphony. Learning will follow three paths: imitating the teacher, reading the score, and emulation among students. Small concerts will be organized to stimulate children and give purpose to their learning. The OC teachers are professional certified string players specifically prepared for the OC methods at the Accademia d’Archi, an accredited music school in Geneva specialized in string instrument education. They will be supervised and coached by the director of the Accademia d’Archi (SNSF scientific partner). Every three months, a video of the OC courses will be recorded. A short video of each individual child playing the same short piece will also be made. The videos allow rating the playing level and progress post hoc by independent professional string players.

##### Experimental intervention group 2: VA

Diverse forms of VA will be taught. There will be two certified art teachers per class, to ensure sufficient individual attention. The courses will center on four axes: collage, drawing, painting, and sculpting, to offer a varied and attractive program, but may include other activities from the visual arts. The four different activities take place in blocks of ~6-8 weeks. Like for the OC interventions, working on individual aspects such as line, shape, form, color and tone will be part of the interventions for each axis, but the goal is to produce holistic artworks. At the end of each axis, the final task consists of the autonomous creation of an original artwork by the child. After approximately one month, the children can take some materials home and are encouraged to do artwork for 20 minutes a day, five days per week in the 1^st^ year, and 30 minutes in the 2^nd^ year, verified via an online diary. Small exhibitions will be organized to stimulate children and give purpose to their learning. Every three months, a video of the VA courses will be recorded. A short video of each individual child working on or presenting their artwork will also be made. This, as well as photographs of the artworks, will allow independent art teachers to rate the children’s skill level and progress post hoc. The VA teachers are professional artists specifically prepared for the VA courses under the supervision of a specialist in child art education (SNSF scientific partner) of the HEP (Haute École Pédagogique) de Lausanne.

#### Active Control: group CG

In order to ensure ethical recruitment and reduce motivational bias, we will not include a passive control group. Instead, we will establish an active control group that participates in cultural outings approximately every six weeks, aligned with our participants' interests and age range. These outings include attending orchestra rehearsals, visiting a marionette theater, exploring botanical gardens, or visiting museums.

These outings are expected to have minimal impact on the development of executive functions and brain plasticity, allowing the active control group to mirror natural development. Nevertheless, these cultural outings may enhance creativity, social skills and emotional well-being, while also serving as a controlled measure of social interaction compared to the artistic interventions [79].

#### Intervention fidelity

To ensure intervention fidelity^2^, we will establish procedures defining stages of learning. For both interventions, sessions will be organized with the teachers under the supervision of experienced art professors in the respective fields, and well acquainted with the targeted age group. To guarantee the quality of the courses, teachers must hold at least a Bachelor's degree or be enrolled in a Master's program. Additionally, they should have prior experience in teaching children in a classroom setting. Clear learning steps will be identified. Supervisors and teachers will meet regularly to discuss difficulties. Supervisors will visit the courses twice in the first three months and thereafter every three months to ensure streamlined and coherent interventions.

### Study population

We intend to enroll 150 school children on a voluntary basis, with the goal of initially having 50 participants in each group (for detailed power analyses, refer to section "Statistical analysis plan and sample size calculation"). The courses and required materials come at no cost, which may encourage low-income families to participate.

#### Demographic questionnaire, extracurricular activities, and school grades

All families desiring to participate should consent to fill in a demographic questionnaire, comprising questions on the family's socio-economic background, and potential extra-curricular activities of the children. They should also consent beforehand that the research team collects the school grades. They will be informed that all data will be coded, and personal information strictly protected (refer to Declarations section, subsection Availability of data and materials). All these consents will be gathered in one single consent form, composed according to the standards of Swiss ethics (https://swissethics.ch).

Upon agreement with their child, parents will sign the informed consent before participating in the study.

For the age choice justification and motivation, see section "Rationale and overall goals of the project and its interventions".

Inclusion and exclusion criteria are provided in Table 1.

**Table 1:** Inclusion and exclusion criteria

**Inclusion criteria**		
School grade 2P/3P/4P^a^	□ Yes	□ No
Right-handedness [80, 81]	□ Yes	□ No
Sufficient Mastery of the French Language	□ Yes	□ No
Able to give oral informed consent (child)	□ Yes	□ No
Able to give written informed consent (parent)	□ Yes	□ No
**If No, exclude**		
**Exclusion criteria**		
Non-consent (children and or parents)	□ Yes	□ No
Repeated a class with respect to standard curriculum	□ Yes	□ No
Not corrected/severe hearing deficits	□ Yes	□ No
Not corrected/severe vision deficits	□ Yes	□ No
Left-handed or ambidextrous	□ Yes	□ No
Severe neurodevelopmental disorders (e.g. severe dyslexia, severe ADHD)	□ Yes	□ No
Younger than 6 in the month following the beginning of interventions	□ Yes	□ No
Older than 8 in the month preceding the beginning of study interventions	□ Yes	□ No
Protocolled music instrumental practice in the preceding year	□ Yes	□ No
Protocolled visual arts courses in the preceding year	□ Yes	□ No
MRI incompatibility (physical or psychological)	□ Yes	□ No
Psychometric battery incompatibility (physical or psychological)	□ Yes	□ No
**If Yes, exclude**		

Nota Bene: Children unable to pass the psychometric battery (lack of concentration or unable to perform the tasks) or the MRI battery (unable to lay still or perform the fMRI task) at baseline will be excluded from further participation. This will be clearly mentioned in the informed consent.

^a^2P/3P/4P correspond to 5-to-8 YOA in the Swiss public school system. A child enters in 3P if it turns six before July 31 of that school year. 2P, 3P, and 4P in the Swiss system correspond to Grades 2, 3 and 4 of USA Elementary school.

#### Randomization procedure

To assure homogeneity of the groups at the start, we will apply a stratified randomization into OC, VA and CG groups, via a stratified procedure according to the factors 1) age, 2) gender 3) socio-economic background. We will apply a clustering procedure, to obtain the closest triplets of participants, based on the three stratification factors. Then we will randomly attribute one child of each triplet to OC, VA or CG groups. Stratified randomization may increase statistical power while retaining the benefits of random allocation [82]. We applied very similar randomization methods in the past [83].

We will not reveal our specific hypotheses regarding the various interventions to the families and art teachers. Instead, the research will be presented as an assessment of child development following different art courses and cultural outings, which accurately reflects the study's nature.

If we receive more applications than necessary, we will attempt to achieve gender balance. However, as no scientific documentation exists that boys or girls would be advantaged for music practice or visual arts learning, an imbalance of gender is not a major issue. Following the stratified randomized procedure, girls and boys will be evenly distributed over the groups.

### Recruitment, screening, and informed consent procedure

We will recruit 150 school children on a voluntary basis, aiming to initially include 50 in each group (for power analyses, refer to section "Statistical analysis plan and sample size calculation"), authorized by the General Directorate of Obligatory Education (Direction Générale de l'Enseignement Obligatoire (DGEO)). Several primary schools have agreed to participate and will promote the study within their institutions using informational brochures. We will expand our recruitment efforts through various channels, including social media platforms such as Facebook and Instagram, RTS (Radio Télévision Suisse - Swiss TV and Radio), local newspapers, parents’ associations, and the Geneva Foundation for Socio-Cultural Animation, among others.

Parents and children can receive extensive information during drop-in sessions in public venues, ask questions, and register to be contacted thereafter. The families should accept beforehand to be allocated randomly to one of the three groups, OC, VA or CG, and anticipate pursuing the study for the full two years, although they may withdraw at any time without explanation. Additionally, they must consent to MRI recordings and the filming of courses. They should also consent that the team may collect the school grades. The courses are free, which may encourage low-income families to participate.

Parents will receive information that ensures them that all data on their children will undergo coding and their children’s personal information will be rigorously safeguarded (refer to Declarations, subsection Availability of data and materials).

The recruitment period for wave 1 will extend from December 2023 to March 31, 2024. Recruitment will then continue until August 31, 2024, for wave 2. We will start interventions and outings at the end of March 2024 for wave 1. The start of interventions for wave 2 is planned for September 2024. In case of insufficient participant numbers for wave 2, we may extend the recruitment period and start the interventions later.

The investigators will explain to each child participant and their parent(s) the nature of the study, its purpose, the procedures involved, the expected duration, the potential risks and benefits and any discomfort it may entail. Each child participant and their parent(s) will be informed that participation in the study is voluntary, and that the child participant may withdraw from the study at any time.

All potential child participants and their parent(s) will be provided with a participant information sheet and a consent form describing the study and providing sufficient information for participants to make an informed decision about their participation in the study.

The formal consent of a child participant and their parent(s) or guardian(s), using the approved consent form, will be obtained before the participant is submitted to any study procedure.

The consent form will be signed and dated by the investigator or his designee at the same time as the participant signs. A copy of the signed informed consent will be given to the parents of the study participant. The consent form will be retained as part of the study records.

After the information on the study has been provided by one of the communication means mentioned above (see first paragraph of this section (Recruitment, screening, and informed consent procedure)), and once informed consent has been obtained, the initial screening will be done via the demographic questionnaire that the parents should complete. This questionnaire will be sent or given to the parents after signing the consent form and should be filled in after a delay of one week and returned to the research team. Alternatively, we will fill it in with the families at the public venues.

The consent form clearly mentions that one out of three children will end up in the culture group, participate in cultural outings, and participate in all measurements, but will not receive art lessons. In case of non-acceptation to the assigned group, the child cannot participate.

We will not include vulnerable participants, except for their young age. All children not fit for MRI or behavioral testing for physical or psychological reasons will be excluded beforehand. This will be mentioned in the consent form.

The benefits for the children in the OC and VA groups consist of free quality art lessons over two years, as well as a string instrument or visual arts materials to take home for the full intervention period for homework. The control group will participate in engaging cultural outings approximately every six weeks (six times per year). Small gifts will be offered to all children after each measurement session.

### Study procedures with timelines

The planning of the study procedures and its timelines are presented in Table 2.

**Table 2:**
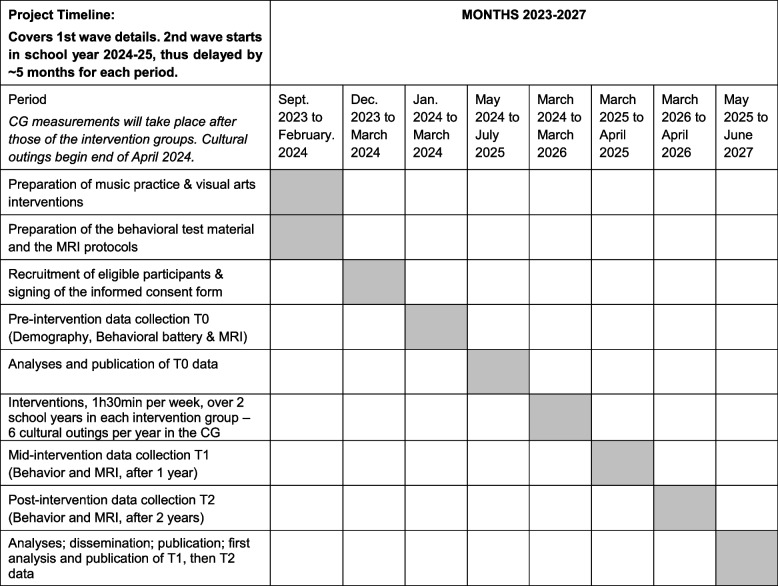
Study procedures with timelines

Baseline (T0) testing will begin approximately six weeks before the onset of the interventions for both art groups. All intervention groups will receive 1 hour and 30 minutes of art lessons per week, during two full years, outside school hours. The mid-intervention testing will take place after one full year of interventions. Final testing will take place after two years of interventions.

Total duration for an average child to participate in the study will therefore take approximately two years and three months (may vary as a function of the dates of screening and testing before and after the interventions), within a period stretching from December 2023 till end of August 2026, depending on which wave the child participates in.

We will cope with test-retest effects by 1) using different versions, or different items, or different order of items of the tests at T0 (baseline, before the interventions), at T1 (after one year of interventions) and at T2 (after two years of interventions), and 2) by considering the test-retest effects in the control group between time points. This also holds for the fMRI visual and auditory WM tasks.

For the behavioral battery, we will adhere to the rule of two tests for each assessed function, primarily choosing tests that have been validated in our target population.

At each time point (T0. T1, T2) a behavioral battery (2x60 minutes at different days) and an MRI battery (45 minutes of measurement, plus 45 minutes of preparation) will be passed by each child.

The behavioral battery primarily focuses on EF, with a special emphasis on WM as the main outcome. Testing of all EF functions is done via at least two different tasks. However, we will also evaluate emotion regulation (ER), which is supposed to be closely associated with EF performance [84–86]. One test on prosocial tendencies [84] is also included. ER and prosocial behavior may be reinforced by group training. We will also evaluate potential direct transfer from the group art courses: musical (OC) [87, 88] and visuospatial capacities (VA) [89].

Finally, we will also employ the International Matrix Test [90], to assess speech in noise perception abilities, which are known to improve with musical training in children [43, 91]. Improvements in speech perception, especially in noisy environments, can significantly enhance school performance and everyday communication [43], thereby positively influencing daily life functioning and academic success.

To avoid order effects, the different tests in the behavioral battery are carried out in two distinct sequences, counterbalanced between the children in each group. The alternation of difficult and simple tasks is balanced in each sequence.

The detailed behavioral and MRI batteries are depicted in Tables 3 and 4.

**Table 3:** Behavioral data collected at all time points. Total testing duration 2*60 minutes, without the fMRI tests and judgements/ratings. It comprises testing of all main EF components using mainly the WISC: Wechsler intelligence scale for children–5th Edition [89] and the KiTAP: Test of Attentional Performance for children [92]

COGNITIVE DOMAIN	FUNCTION	NAME OF THE TEST	BRIEF DESCRIPTION	REF.
Music abilities	Rhythm perception	Intermediate Measures of Music Audiation (IMMA)	Pattern comparison, judge if same or different in rhythm RIGHT button for identical patterns, LEFT for different patterns	[87, 88]
Visuo-spatial abilities	1. Visuo-spatial reasoning & praxis 2. Problem solving	Cubes test (WISC-V)	Arranging physical pictured cubes in specific patterns according to a design	[89]
Working memory (fMRI)	Auditory tonal working memory	Pattern comparison (inspired by IMMA)	Compare two 3-note sinus tone patterns 1. Low load condition (3 identical notes): RIGHT button for identical patterns, LEFT for different patterns 2. High load condition (3 different notes): RIGHT button for identical patterns, LEFT for different patterns	Adapted from [87, 88]
	Visual-spatial working memory	Visual n-back	6-case spatial matrix containing appealing monster figures - conditions: 0-back, 2-back Right button for matched n-back trial l, LEFT button for unmatched n-back trial	[93]
Memory Systems	Short-term memory	Digit Span Forward (WISC-V)	Repeating series of digit sequences of increasing length aloud	[89]
	Working memory: information manipulation	Digit Span Backward (WISC-V)	Repeating series of digit sequences of increasing length aloud backwards	[89]
Attention	Processing speed	"The Witch" (KiTAP)	Specific key for witch presented in window – respond as quickly and correctly as possible	[92]
	Selective and sustained attention	Ucancellation (child-friendly version of d2 test)	Respond to upright dog (tail left), upside-down monkey (tail right) - Ignore similar distractors	[94, 95]
Auditory selective attention	Speech in noise perception	International Matrix Test	Participants repeat 5-word sentences heard through headphones: first without, then with a white noise background (right ear – left ear – both ears)	[90]
Inhibition	Response inhibition (motor inhibition)	Go/NoGo – "The Bat" (KiTAP)	Specific key for bat only - Ignore cat	[92]
	Distractor inhibition (perceptual/cognitive inhibition)	Child-friendly Flanker Task	Left/Right arrow for center fish swimming direction - ignore surrounding fishes (congruent vs. in- congruent swimming direction), as quickly and correctly as possible	[96]
Cognitive control / flexibility (higher EF)	Perceptual switching (simple)	"The dragons' house" (KiTAP)	Match button press to dragon color as quickly and correctly as possible – 2 alternating colors	[92]
	Rule switching (complex)	Dimensional Change Card Sort (DCCS)	Sort cards based on changing rules (color, shape), separately and mixed, as quickly and correctly as possible	[97]
Reasoning (higher EF)	Abstract/logical	Matrix reasoning (WISC-V)	Inspect incomplete matrix – choose missing piece from 5 options	[89]
	Quantitative/analogical	Figure weight (WISC-V)	Inspect scale with missing weight – select balance option within time limit	[89]
Reasoning Daily life functioning	Executive functions	BRIEF questionnaire	Daily living assessment of children’s executive functions	[98, 99]
Learning and progress	N/A	Multi-media materials	1. Videos (orchestra) – rating on ≠ Likert scales by independent judges 2. Photos and videos (visual arts) – rating on ≠ Likert scales by independent judges	
Learning and progress	N/A	Teacher ratings (Likert scales)	Music: 1. pitch; 2. rhythm; 3. expression; 4. ensemble playing/singing; 5. motivation; 6. overall quality Visual Arts: 1. graphic skill; 2. color management; 3. expression; 4. originality; 5. motivation; 6. overall quality	
	N/A	Homework	Use an online app to track homework	
Others / Miscellaneous	N/A	Aerobic fitness	School gymnastic teachers report aerobic fitness on Likert scale	
	N/A	Demographic questionnaire	Questionnaire for parents (extensive, including extracurricular activities)	
	N/A	Academic results	Collection of all school grades	
	Emotion regulation	Emotion Regulation Checklist French Version (ERC-vf)	French version of questionnaire concerning children; 24 questions on a 4-point Likert scale measuring 2 factors: emotion regulation & emotional lability	[85]
		Emotion Regulation Questionnaire for Children & Adolescents (ERQ-CA)	French version of questionnaire for children (self-report); 20 questions on a 5-point Likert scale measuring 2 strategies: cognitive reappraisal and expressive suppression	[100]
	Behavioral and emotional problems	Strengths and Difficulties Questionnaire (SDQ_Fra)	French version of questionnaire concerning children; 25 questions on a 3-point Likert scale measuring 3 factors: internalizing problems, externalizing problems, and prosocial tendencies	[84]

**Table 4:** Brain magnetic resonance imaging (MRI) test battery. The total recording duration is < 45 min. (fMRI: functional MRI; MP2RAGE: magnetization-prepared 2 rapid acquisition gradient echoes; FOV: Field Of View; TR: Time to Repetition; TE: Time to Echo; TI1: Inversion Time 1; TI2; Inversion Time 2; EPI: Echo Planar Imaging)

**Type of structural/functional MRI**	**Sequences (total duration < 45:00 including technical sequences)**
*Gray matter assessment* Thickness, surface area, volume changes [101]	T1-weighted MRI, MPRAGE [*05:12*]; voxel size: 0.8 mm isotropic; 208 slices; FOV: 230x230x166.4 mm; GRAPPA acceleration factor: 2; TR/TE: 2000/2.49 ms; TI: 900 ms; flip angle 9 degrees
*White matter assessment* Evaluation of the structural connectivity: white matter fiber connections features (integrity, diffusivity etc.), crucial for information exchanges between different parts of the brain ([102]	Multishell Diffusion Weighted Imaging (DWI) [*07:12*]; voxel size: 1.8 mm isotropic; 76 slices; FOV: 208x208x120 mm; GRAPPA acceleration factor: 2, multiband accelerator factor: 2; TR/TE: 3573/61.6 ms; b-values: 0/1000/2000 s/mm^2^; 9/30/60 directions, anterior to posterior phase encoding direction followed by a reverse direction for distortion correction; b-values: 0/ s/mm^2^)
*Task-related brain activity (task-fMRI:* 1. Tonal WM adapted from [87, 88]	1 EPI run [≈*09:30*]; voxel size: 2.5 mm isotropic; 51 slices; FOV: 210x210x127.5 mm; TR/TE: 1000/30 ms; multiband accelerator factor: 3
2. Visual n-back WM, adapted from [93]	1 EPI run [*≈09:30*]; voxel size: 2.5 mm isotropic; 51 slices; FOV: 210x210x127.5 mm; TR/TE: 1000/30 ms; multiband accelerator factor: 3
*Resting State brain activity (RS-fMRI)* Measures activity at rest and global functional connectivity of the brain (temporal correlations of activity between distant regions) [103]	1 EPI run [*08:30*]; voxel size: 2.5 mm isotropic; 51 slices; FOV: 210x210x127.5 mm; TR/TE: 1000/30 ms; multiband accelerator factor: 3

### Withdrawal and discontinuation

As mentioned in the informed consent form, children and their parents can decide to withdraw from the study at any time without providing a justification.

If there is a suspicion of sudden cognitive impairment, psychological distress, or MRI incompatibility, the parents of the child will first be informed. Whether the child participant may pursue the interventions, if they so wish, will be determined on an individual basis by the parents, their family doctor, the schoolteacher and the school director, in agreement with the principal investigator of the study.

## Statistics and methodology

### Statistical analysis plan and sample size calculation

The research statistician of the host institute of the principal investigator will assist with the behavioral analyses. Experts in MRI data analysis, machine learning, and multimodal data integration within the team will supervise both MRI data analysis and the combined multivariate analysis of behavioral and MRI data.

*Sample size*: We computed sample size separately for our main behavioral outcome (WM) and for fMRI and structural MRI data. Our sample size will consist of 50 children per group, and we account for an attrition of 30%, so for the final analyses, we will dispose of minimum 35 children per group.

For our main behavioral outcome WM: Based on our previous behavioral study on OC [1]: with an alpha=0.05 and power=0.80, the projected sample size needed to reach a similar effect size (GPower 3.1) *n*=48 is the total for a simple between-group comparison, thus *n*=24 per group.

For fMRI data: A cross-sectional study comparing visual working memory networks of two groups of 19 ADHD and 14 healthy children (8-12 YOA) using a similar design [104] obtained activation maps within groups and between groups with numerous clusters surviving to correction for multiple comparisons at *p*<0.05 FWE (family-wise error).

For structural MRI data: A study showed large developmental brain plasticity changes in gray/white matter volume and fractional anisotropy (FA) at the whole-brain level (*p*<0.05 corrected for multiple comparisons) using a longitudinal design in a group of 24 adolescents [105].

To address the issue of selective dropout, we adjusted our participant numbers to account for an anticipated attrition rate of 30%, while still meeting the necessary sample size criteria, computed above.

A longitudinal randomized controlled trial (RCT) with three measurement time points and sufficient sample sizes offers increased statistical power and helps mitigate the risk of false positive findings commonly associated with smaller, non-randomized studies.

#### Planned statistical analyses

Step 1: we will study relationships between EF and brain data collected at baseline for all participants based on a priori hypotheses and also in an exploratory way, using repeated measures Analysis of Covariance (ANCOVA), general linear models (GLM), correlations, and regression analyses. Apart from verifying a priori hypotheses, an additional goal is to identify potential confounding factors affecting our main outcome: EF, and particularly WM performance. Those confounding factors include socio-economic status, extra-curricular activities, aerobic physical fitness, and sleep. Apart from studying the influence of these factors on the outcomes, they also allow nuisance factor control, which will allow to objectively evaluate the OC and VA impact on EF in the following steps.

Brain data will be pre-processed and analyzed using in-house script routines calling the last versions of the standard neuroimaging software pipelines (e.g. Statistical Parametric Mapping 12 (or later version if released; https://www.fil.ion.ucl.ac.uk/spm/). We will generate a population-specific brain template. Individual brains and activation maps will be normalized on this template, critical for the validity of longitudinal brain plasticity results [106].

Step 2: we will examine and compare the development in both experimental groups and the CG over time for the comprehensive set of EF and brain data separately (T0 vs. T1 vs. T2). We will use linear mixed models (LMMs) with the open source software R [107] to evaluate the longitudinal effects for all behavioral variables separately, comparing all three groups over two years. LMMs are flexible, powerful, statistical models that can consider several levels of clustering, continuous and qualitative explanatory variables, as well as unbalanced data (pertinent if attrition differs per group). For testing specific hypotheses, we will add specific brain data in those LMMs (f.i. WM associations with CEN functional connectivity).

Near transfer scores closely representing direct benefits from the art interventions on the trained domains (musicality: IMMA [86, 87], visuo-spatial aptitude: the cubes test [84], teacher’s judgements, ratings by independent judges; see Table 3) will be analyzed in the same way. The results allow evaluating individual learning and the effectiveness of the courses.

For the brain data we will use univariate and multivariate analysis methods. Individuals’ intensity of training, progress, demographic characteristics, physical aerobic fitness and appreciation of the courses will be considered.

Step 3: we will analyze all data in a multivariate data-driven thus exploratory way. Separately analyzing behavioral data and several kinds of brain imaging data, cross-sectionally and over time, provides extremely valuable information on these data. Additionally, combining diverse behavioral and multimodal brain MRI data within data-driven multivariate analyses may unravel hidden “covert” relationships [108, 109]. These advanced analyses will encompass two main axes. The 1^st^ axis will be concerned with group inferences, asking “what intervention-related changes can be observed on imaging data at the group level?”, while the 2^nd^ axis will be concerned with individual prediction: “Can imaging features predict behavioral outcomes at the individual level?” To this end, we will consider joint analyses of imaging data (structural connectivity, functional connectivity data considered together).

In the 1^st^ axis (group-level analysis), we will use two methods which have been used successfully on multimodal brain imaging data, each carrying different assumptions about linearity of relationships and distribution of variables: multimodal Independent Component Analysis (ICA) [108, 109] on the one hand, and Partial Least Squares (PLS) [110–112] on the other. We detail these methods below.

1. Joint, fusion, and linked ICA [113, 114] are variants of ICA that allow combining different types of data such as functional and structural neuroimaging data. These analyses could reveal joint changes in different imaging modalities (i.e. covariations in functional and structural connectivity measures) and allow to investigate differences between our three groups [108, 113].
2. PLS is another powerful technique that can identify components of multivariate relationships between imaging and behavioral data [111, 115], notably PLS correlation models [111]. PLS and sparse PLS may reveal hidden relationships between battery tests and MRI measurements before and after the regimens (OC and VA) and show whether these relationships differ between the groups. Implementations of these approaches are publicly available in addition to several in-house extensions that allow using them in the most flexible way, including machine learning [74, 116].

**Fig. 1:**
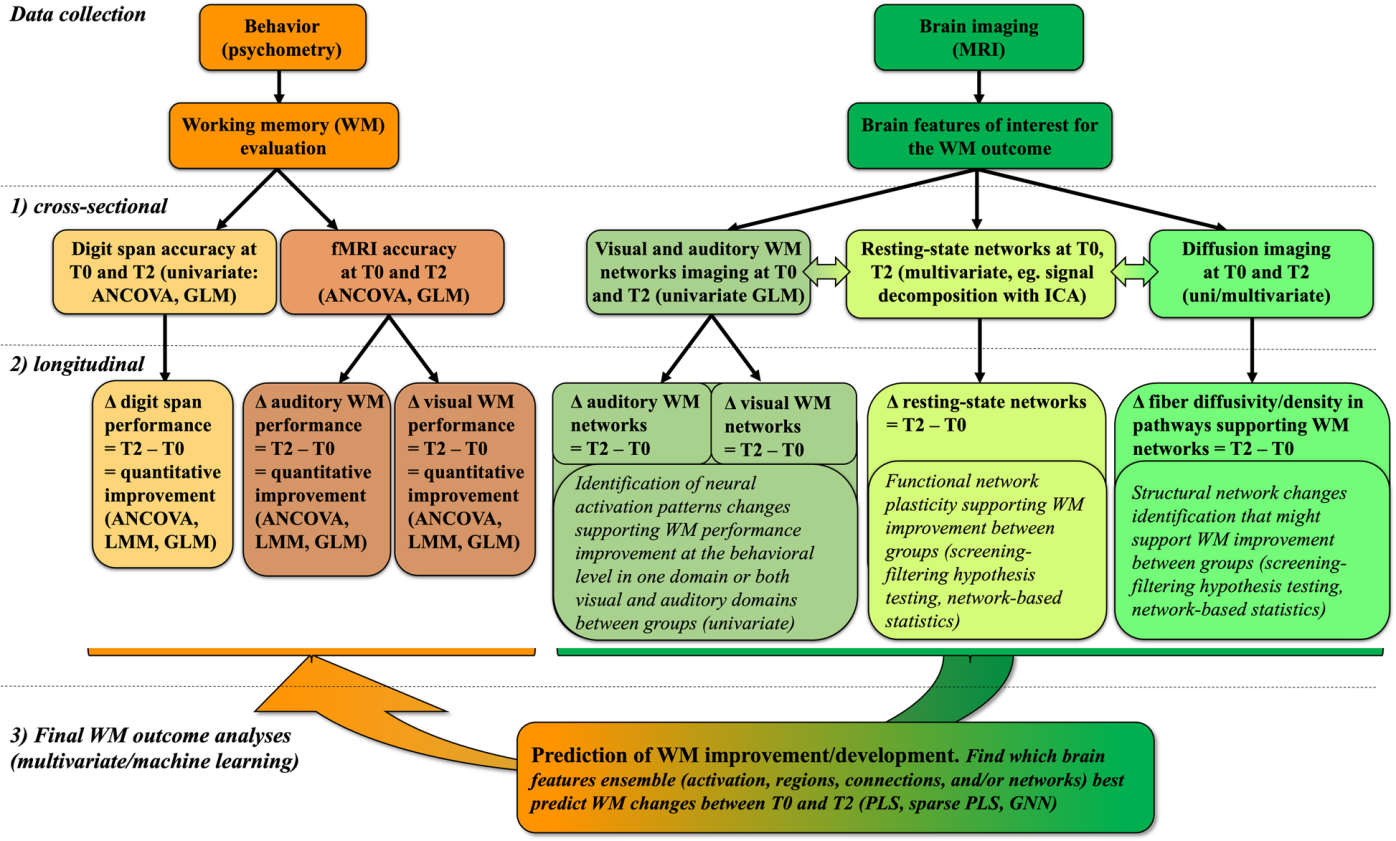
Data analysis overview This table summarizes steps and methods used to achieve our ultimate goal i.e., the prediction of the main behavioral outcome: working memory improvement and development (WM: working memory; ANCOVA: Analysis of Covariance; LME: linear mixed model equations; GLM: general linear model; MRI: magnetic resonance imaging; ICA: independent component analysis; ICAPs: innovation-driven co-activation patterns, PLS: partial least squares; GNN: graph neural networks)

In the 2^nd^ axis (individual prediction), we will attempt to predict the difference in behavioral outcomes (digit span, auditory/tonal, and visual WM) from the difference in connectomes^3^ between time points T0 and T2. The control group will allow discarding developmental connectivity differences that are not related to our interventions or not meaningful for predicting working memory. Here, we will leverage recent advances in graph-based machine learning, particularly graph neural networks (GNN). We will use each subject’s connectivity values (either in the whole brain, or in specific functional networks of interest (CEN, SN)) as input to train a GNN model to predict the behavioral scores. The scores can be predicted one by one, or jointly by using a multi-output network (see Fig. 1 for an overview of the analyses). To improve performance, we will use a technique called *transfer learning*, by pre-training the GNN model using related tasks and scores from other cohorts, and then fine-tune using our own data. The team of Richiardi successfully developed related transfer learning approaches on other datasets. Further, we will use our newly developed graph data augmentation technique^4^ to ensure that sample size is not an issue. Using a GNN model allows us to perform this analysis for both the structural and functional connectomes, where functional connectomes are obtained either from resting-state or from task-based connectivity.

Significance level will be strictly controlled using false discovery rate special techniques for multiple comparison correction for univariate and multivariate analyses involving behavioral, MRI and combined behavioral and MRI data [117].

### Handling of missing data and drop-outs

#### Handling of missing data

Common approaches, like substituting missing data by the group mean, induce the risk of reducing statistical variability and study validity. In consequence, we will impute missing values using the regularized iterative Principal Component Analysis (PCA) algorithm developed by Josse, Husson and Pagès [118], implemented in the R software.

We will prevent drop-outs as much as possible by verifying at T0 the capacity of the child to pass the behavioral tests and MRI scans (psychological or physical problems). If a child fails to meet the requirements of the psychometric tests and/or MRI measurements, they will not be eligible to participate. This exclusion will be explicitly stated in the informed consent form.

We have multiple means to assess and facilitate children's adaptation to the MRI environment: 1) we prepared a video of a child undergoing MRI scanning in the BBL for the families to watch beforehand, 2) the BBL's virtual reality facility, "MRI Adventure", may prepare the children for the MRI setting, and 3) a mock scanner at the principal investigator's host institution will help the children to evaluate their comfort in the MRI environment.

#### Attrition / drop-outs

Ultimate target: a total of at least 35 participants at T2 in each group (after the 2-year interventions)

Anticipating an attrition of 30% we will recruit approximately 150 participants, allowing to reach the final number of 35 individuals per group, which is necessary to reach sufficient statistical power (see section "Statistical analysis plan and sample size calculation").

- 1^st^ experimental group Orchestra in Class OC: *n* = 50
- 2^nd^ experimental group Visual Arts: *n* = 50
- Active control group "Culture" CG: *n* = 50
- Total number of recruited participants ready to participate: 150

## Discussion

### Feasibility of the study concerning the (f)MRI part

#### Timing of the MRI recordings

Recruitment, MRI, and behavioral data collection for wave 1 will run from December 2023 to the end of March 2024. Interventions will begin at the end of March 2024. Wave 2 is delayed by approximately five months, with interventions starting in September 2024. We benefit from privileged access to the MRI facility from the Brain and Behaviour Laboratory (BBL), fixed hours on weekdays, as well as after-hours and weekends. We will capitalize on holidays as well, which will allow us to reach our data collection target of 10-15 children per week.

#### Experience with the targeted age group

Our team has a large experience in child behavioral and neuroimaging studies. This includes past research involving high-density electroencephalogram (EEG), MRI and behavioral data collection in children aged 6-12 years by various team members [1, 46, 119–122].

#### Consent and exclusion criteria

Consent to participate in MRI recordings is a prerequisite for inclusion in the study. Our preparatory measures, such as the "MRI adventure" at the University of Geneva’s BBL, or a mock scanner experience, will help assess each child’s suitability for MRI recordings. Children who are not suited for MRI due to various factors will be excluded, as outlined in the consent forms (f.i. claustrophobia, anxiety, incapability to perform the fMRI tasks, impossibility to lay relatively still for 45 minutes (in between sequences, the children can move their body, hands and feet)).

### Attrition

In response to the concern of selective dropout, we revised our participant numbers to accommodate an expected attrition rate of 30% while maintaining the required sample size criteria (refer to section "Statistical analysis plan and sample size calculation" for details).

### Statistical power

A longitudinal randomized controlled trial (RCT) featuring three measurement time points and adequate sample sizes provides greater statistical power and reduces the likelihood of false positive findings often seen in smaller, non-randomized studies. The baseline measures (T0) will be conducted before the interventions, allowing us to replace any child unable to complete the psychometric or MRI assessments. Additionally, any discovery of brain anomalies during the T0 MRI will lead to exclusion from further participation, with appropriate steps taken to inform parents and physicians.

### Strengths of this study

This is the first RCT examining the influence of two distinct intensive art interventions on cognitive and cerebral development in young children compared to a control group.

The study focuses on children of all socioeconomic backgrounds with an optimal age for brain and behavioral plasticity.

### Limitations of this study

Recruiting children and their families who accept and respect the intensive intervention schedule and measurements, particularly brain imaging, will be challenging. We will handle this issue by providing extensive information to the families, also considering their cultural backgrounds.

Participant attrition is a main risk, that we will mitigate by implementing intensive communication strategies involving families, the scientific team, and the art teachers (also see Attrition). A “hotline” for questions and remarks will be open all week during office hours.

Participation in the study does not restrict parents from seeking additional enriching activities for their children during the study.

## Conclusion

In summary, this study protocol is designed to investigate the influence of artistic and cultural activities on children's cognitive and brain development at the beginning of their academic journey. It adopts a multidisciplinary approach that combines enriching educational techniques with neuroscientific perspectives. The potential outcomes hold promise for enhancing teaching methods and expanding our comprehension of children's cognitive and brain development, both with and without exposure to artistic enrichment. The overarching goal of our research is to develop strategies that can positively impact the well-being and future potential of all children. This study represents a unique and valuable effort to improve children's development and well-being by merging evidence-based educational practices with a deeper understanding of the intertwined underlying processes of brain and cognitive development.

### Supplementary Information

**Supplementary Material 1.** 

## Data Availability

No datasets were generated or analysed during the current study.

## Abbreviations

(f)MRI: (functional) Magnetic Resonance Imaging
ADHD: Attention Deficit Hyperactivity Disorder
ANCOVA: Analysis of Covariance
BASEC: Business Administration System for Ethics Committees
BBL: Brain and Behaviour Laboratory
BRIEF: Behavior Rating Inventory of Executive Function
CCER: Commission Cantonale d’Éthique de la Recherche de Genève / Geneva Canton Ethics Commission
CEN: Central Executive Network
CG: Control Group
DCCS: Dimensional Change Card Sort
DGEO: Direction Générale de l’Enseignement Obligatoire / General Directorate of Obligatory Education
DMN: Default Mode Network
DWI: Diffusion Weighted Imaging
EEG: Electroencephalogram
EF: Executive Functions
EPI: Echo-Planar Imaging
ER: Emotion Regulation
ERC_vf: Emotion Regulation Checklist French Version
ERQ-CA: Emotion Regulation Questionnaire for Children & Adolescents
FA: Fractional Anisotropy
FC: Functional Connectivity
FEW: Family-Wise Error
FOV: Field Of View
GEMMI lab: Geneva Musical Minds laboratory
GLM: General Linear Model
GNN: Graph Neural Network
GRAPPA: GeneRalized Autocalibrating Partial Parallel Acquisition
HEdS-GE: Haute École de Santé de Genève HES-SO / Geneva School of Health Sciences
HEP: Haute école pédagogique de Vaud
HES-SO: Haute École Spécialisée de Suisse occidentale / University of Applied Sciences and Arts Western Switzerland
HUG: Hôpitaux Universitaires de Genève / Geneva University Hospitals
ICA: Independent Component Analysis
iCAPs: Innovation-driven Co-Activation Patterns
IMMA: Intermediate Measures of Music Audiation
IMMA: Intermediate Measures of Music Audiation
IQ: Intelligence Quotient
KiTAP: Testbatterie zur Aufmerksamkeitsprüfung – Kinderversion / Test of Attentional Performace – child-friendly version
LME: Linear Mixed Model Equations
LMM: Linear Mixed Model
MIP: Musical Instrumental Practice
MP2RAGE: Magnetization-Prepared 2 Rapid Acquisition Gradient Echoes
MRI: Magnetic Resonance Imaging
NCT: National Clinical Trial
OC: Orchestra in Class
PCA: Principal Component Analysis
PLS: Partial Least Squares
RCT: Randomized Controlled Trial
RS-fMRI: Resting State functional Magnetic Resonance Imaging
RTS: Radio Télévision Suisse / Swiss TV and Radio
SDQ_Fra: Strengths and Difficulties Questionnaire (French version)
SN: Salience Network
SNCTP: Portal for clinical trials in Switzerland
SNSF: Swiss National Science Foundation
T0: Baseline measurement
T1: One-year mid-intervention measurement
T2: Two-year post-intervention measurement
TE: Time to Echo
TI1: Inversion Time 1
TI2: Inversion Time 2
TR: Time to Repetition
VA: Visual Arts
WISC-V: Wechsler Intelligence Scale for Children – 5^th^ edition
WM: Working Memory
YOA: Years Of Age

## Footnotes

1 Cluster RCT: entire classes were randomly assigned to the music practice or the active control group. Consequently, individual children did not have a choice in participating in OC interventions, but they were not individually randomized.

2 Ensures the study is conducted as planned.

3 A connectome describes a comprehensive set of neuronal connections in the central nervous system

4 Mazen Mahdi, Graph Representation and Inference for Individual Gray Matter Networks from Structural Magnetic Resonance Imaging, EPFL, unpublished Master Thesis, 2020
