# Correction: Cognitive enrichment through art: a randomized controlled trial on the effect of music or visual arts group practice on cognitive and brain development of young children

**DOI:** 10.1186/s12906-024-04504-3

**Published:** 2024-05-28

**Authors:** C. E. James, M. Tingaud, G. Laera, C. Guedj, S. Zuber, R. Diambrini Palazzi, S. Vukovic, J. Richiardi, M. Kliegel, D. Marie

**Affiliations:** 1 Geneva School of Health Sciences, Geneva Musical Minds lab (GEMMI lab), University of Applied Sciences and Arts Western Switzerland HES-SO, Avenue de Champel 47, Geneva, 1206 Switzerland; 2 Faculty of Psychology and Educational Sciences, University of Geneva, Boulevard Carl‑Vogt 101, Geneva, 1205 Switzerland; 3 Center for the Interdisciplinary Study of Gerontology and Vulnerability, University of Geneva, Chemin de Pinchat 22, Carouge (Genève), 1227 Switzerland; 4 CIBM Center for Biomedical Imaging, Cognitive and Affective Neuroimaging section, University of Geneva, Geneva, 1211 Switzerland; 5 Accademia d’Archi. Route de Chêne 153, Chêne-Bougeries, 1224 Switzerland; 6 Haute école pédagogique de Vaud (HEP, State of Vaud), University of Teacher Education, Avenue de Cour 33, Lausanne, 1014 Switzerland; 7 Department of Radiology, Lausanne University Hospital and University of Lausanne, Rue du Bugnon 21, Lausanne, 1011 Switzerland; 8 Brain and Behaviour Laboratory, Centre Médical Universitaire, University of Geneva, Rue Michel-Servet 1, Geneva, 1211 Switzerland

**Correction: BMC Complement Med Ther 24, 141 (2024)**.

10.1186/s12906-024-04433-1.

Following publication of the original article [1], the authors identified an error in the author name of R. Diambrini Palazzi.

The incorrect author name is: R. Diambrini Palazzo.

The correct author name is: R. Diambrini Palazzi.

The author group has been updated above and the original article [1] has been corrected.

## References

[1] James C, Tingaud M, Laera G (2024). Cognitive enrichment through art: a randomized controlled trial on the effect of music or visual arts group practice on cognitive and brain development of young children. BMC Complement Med Ther.

