# Supplementary material for: Cognitive enrichment through art: a randomized controlled trial on the effect of music or visual arts group practice on cognitive and brain development of young children
Source: BMC Complement Med Ther. 2024 Apr 4;24:141. doi: 10.1186/s12906-024-04433-1 (PMC10993461; doi:10.1186/s12906-024-04433-1)
Supplement: Supplementary file 1 — Supplementary Material 1. [file 12906_2024_4433_MOESM1_ESM.docx]

| **Table 1** Trial Registration Data Set (according to WHO guidelines) | |
| --- | --- |
| Data category | Information |
| Primary registry and trial identifying number | ClinicalTrials.gov ; Identifier: NCT05912270 (NCT National Clinical Trial) |
| Date of registration in primary registry | 20.09.2023 (clinicaltrials.gov) ; Identifier: NCT05912270 |
| Secondary identifying numbers | Study ID number: 126645 (clinicaltrials.gov) |
| Source(s) of monetary or material support | Swiss National Science Foundation (SNSF no. 100014_214977) |
| Primary sponsor | Prof. Clara E. James (the Sponsor-Investigator is the Principal Investigator) |
| Contact for public and scientific queries | Prof. Clara E. James : [clara.james@hesge.ch](mailto:clara.james@hesge.ch) |
| Title | SNSF title: Orchestra in Class, a novel booster for executive functions and brain development in young primary school children (Acronym ORBIT)  Running title: Cognitive enrichment through art: a randomized controlled trial on the effect of music or visual arts group practice on cognitive and brain development of young children (acronym Art and Development) |
| Countries of recruitment | Switzerland |
| Health condition(s) or problem(s) studied | Cognitive and brain development in young primary school children with and without intensive artistic interventions |
| Intervention(s) | Experimental group 1: Orchestra in Class (OC): 1h30m per week, over 2 full years (string instruments: violin, viola, cello, double bass; different musical styles)  Experimental group 2: Visual Arts (VA): 1h30m per week, over 2 full years (different axes: e.g. painting, drawing, collage, sculpture) |
|  | Active control group: 6 cultural outings per year over 2 full years (assisting at events such as concerts, visiting museums, botanical gardens, puppet theaters) |
| Key inclusion and exclusion criteria | Ages eligible for study: 6 to 8 years at start  Sexes eligible for study: both Accepts healthy volunteers: only |
|  | Inclusion criteria: School grade 3 or 4^th^ class primary school (6 to 8 years at start); Right-handedness; Sufficient Mastery of the French Language; Able to give oral informed consent (child); Able to give written informed consent (parent). If No, exclude. |
|  | Exclusion criteria: Non-consent (children and or parents); Repeated a class with respect to standard curriculum; Not corrected/severe hearing deficits; Not corrected/severe vision deficits; Left-handed or ambidextrous; Severe neurodevelopmental disorders (e.g. severe dyslexia, severe ADHD); Older than 7 at the beginning of the school year if 3P; Older than 8 at the beginning of the school year if 4P; Protocolled music instrumental practice in the preceding year; Protocolled visual arts courses in the preceding year; MRI incompatibility (physical or psychological); Psychometric battery incompatibility (psychological).  We will not include vulnerable participants, children not fit for MRI or behavioral testing (physical or psychological reasons) will be excluded (mentioned in the informed consent). |
| Study type | Interventional |
|  | Allocation: randomized intervention model according to 3 stratification factors (age, gender, and socioeconomic status). Groups are balanced for those factors. |
|  | Primary purpose: boost executive function and linked cerebral development in young children at the start of their academic career through intensive art interventions. |
| Date of first enrolment | December 2023 |
| Target sample size | 150 |
| Recruitment status | 40 enrolled |
| Primary outcome(s) | Improve working memory performance in children through 2 distinct art interventions, comparing 1-year and 2-year scores to baseline scores (i.e. progress) and to the CG scores (natural development). |
| Key secondary outcomes | 1. Identify significant differences in structural and functional brain plasticity following the 2 art interventions, comparing 1-year and 2-year values to baseline values and to the CG values; 2. Establish significant correlations between brain plasticity and EF performance change, comparing 1-year and 2-year values to baseline values and to the CG values. 3. Collected data of this proposal will allow, using machine learning, to build a data-driven multivariate model of children's interconnected brain and EF development over the first 2 years of their academic curriculum (6-8 years at start), with or without music or other art training. |
